# An academic achievements visualization research since the 21st century: research on salvage surgery for head and neck cancer

**DOI:** 10.3389/fsurg.2024.1378529

**Published:** 2024-04-08

**Authors:** Bo Zhou, Jingyi Cheng, Kexin Deng

**Affiliations:** ^1^ Department of Head and Neck Surgery, Hunan Cancer Hospital and the Affiliated Cancer Hospital of Xiangya School of Medicine, Central South University, Changsha, Hunan, China; ^2^ Xiangya Stomatological Hospital & Xiangya School of Stomatology, Central South University, Changsha, Hunan, China; ^3^ Hunan Key Laboratory of Oral Health Research & Hunan Clinical Research Center of Oral Major Diseases and Oral Health & Academician Workstation for Oral-Maxilofacial and Regenerative Medicine, Central South University, Changsha, Hunan, China; ^4^ Department of Plastic and Reconstruction, The Third Xiangya Hospital of Central South University, Changsha, Hunan, China

**Keywords:** head and neck cancer, academic visualization, salvage surgery, bibliometrics, CiteSpace

## Abstract

**Background:**

Head and neck cancer is the 6th most common malignancy worldwide, and its incidence is still on the rise. The salvage surgery has been considered as an important treatment strategy for persistent or recurrent head and neck cancer. Therefore, we conducted a bibliometric analysis of salvage surgery for head and neck cancer since the 21st century.

**Methods:**

The literature about salvage surgery of head and neck cancer in Web of Science was searched. CiteSpace and VOSviewer were used to analyze main countries, institutions, authors, journals, subject hotspots, trends, frontiers, etc.

**Results:**

A total of 987 papers have been published since the 21st century. These publications were written by 705 authors from 425 institutions in 54 countries. The United States published 311 papers in this field and ranked first. *Head & Neck* was the most widely published journal. The main keyword clustering included terms such as #0 stereotactic radiotherapy (2012); #1 randomized multicenter (2007); #2 salvage surgery (2004); #3 functional outcomes (2014); #4 transoral robotic surgery (2013); #5 neck high-resolution computed tomography (2010); #6 complications (2008); #7 image guidance (2019). The current research frontiers that have been sustained are “recurrent”, “risk factors”, and “reirradiation”.

**Conclusion:**

The current situation of the salvage surgery for head and neck cancer in clinical treatments and basic scientific research were summarized, providing new perspectives for the development of salvage surgery for head and neck cancer in the future.

## 1 Introduction

Head and neck cancer (HNC) is a heterogeneous set of cancers that occur in diverse anatomical sites of upper aerodigestive tract, including oral cavity, sinonasal cavity, pharynx and larynx (1). According to the GLOBOCAN database for 2020, HNC is the 6th most common cancer worldwide, with estimated 930,000 new cases and 470,000 deaths annually (2). The incidence of HNC is continuously rising with an anticipated 30% increase per year by 2030 (3). Among all HNCs, squamous cell carcinoma accounts for approximately 90% as the predominant histological subtype, and the remaining 10% is composed of less common types such as adenocarcinoma (4). Tobacco use and alcohol consumption are the two most important risk factors for HNCs, and most HNCs now have been linked with human papillomavirus (HPV) infection (5).

Treatments for HNC comprise surgery, radiation therapy, chemotherapy, targeted therapy, immunotherapy, or a combination of treatments. As a result of late diagnosis, local recurrence, distant metastasis, and resistance to therapies, despite the variety of available treatment options, the prognosis for HNC patients remains poor with an overall 5-year survival rate of less than 50% (6, 7). Among HNC patients with lymph node metastasis, the 5-year survival rate is decreased by ∼50%. When extracapsular spread of lymph node metastasis occurs, a further sharp drop in survival rate and an extremely high rate of local-regional and distant failure are presented (8). Even with successful remission through treatment, 30%–60% of HNC patients diagnosed at advanced stage will develop into recurrent locoregional cancers or second primary cancers (9). What's more, recurrent HNC has a stronger trends of tumor infiltration and multifocality by reason of prior treatment effects (10). Therefore, how to cope with unsatisfactory treatment outcomes is always a clinical challenge.

Salvage surgery refers to surgical intervention following failed initial treatment in diverse scenarios, including treatment of delayed neck metastasis, recurrent primary tumors, or even lung metastasis (11). Radical surgical resection is the primary treatment of HNC, and non-surgical treatments are also becoming a primary strategy for preserving organ or function. However, 25%–48% of patients relapse after non-surgical therapy (12), and salvage surgery has been considered as a credible treatment for persistent or recurrent disease. Salvage surgery requires extensive resections and flap reconstructions relying on experienced surgical team. Even then, a high associated morbidity and complication rates still may be caused by the toxicities of primary treatments and the difficulties in determining the range of surgical resection. Therefore, whether to perform the salvage surgery is a tough decision, which should take multiple factors such as tumor stage, grade, efficacy of radiotherapy and chemotherapy, patient age and other systemic diseases into consideration, and more research is needed to improve the comprehensive evaluation system. Despite improvements in surgery and radiochemotherapy, salvage surgery remains a challenging path with current success rates generally not exceeding 30% (13). The prognosticators include tumor site, stage, HPV, margins, disease-free interval, etc. (13). In order to achieve better outcomes of salvage surgery, it's crucial to clarify how to define the best candidates, optimize surgical methods and reduce complications.

Salvage surgery has a long history and encompasses numerous research branches, types, and perspectives, being significant for guiding clinical practices. Therefore, we conduct bibliometrics to systematically review the achievements and development directions of the field. Scientometrics is a cross-disciplinary science that uses mathematical and statistical methods to quantitatively analyze all knowledge domains (14). It integrates mathematics, statistics, and literature science, emphasizing quantitative and comprehensive knowledge system, which can explore the characteristics of scientific fields, understand the knowledge structure and emerging trends of research fields (14). Through visual analysis of large amounts of information, the most influential scholars, institutions, and countries can be discovered, as well as the development process and trends of research, the key and frontier trends of research fields (15). With the development of computer information technology, digital technology and visual analysis have supported the reproducibility, open source, and timeliness of scientometrics research (16).

In this paper, we aim to provide readers with a comprehensive and systematic study of scientometrics in the field of salvage surgery for HNC. Specifically, this analysis focuses on key issues such as the cooperation and co-emergence of countries, institutions, authors, and so on. Special attention is also paid to the temporal and spatial changes in the development priorities and frontiers of the whole field. At the present paper, CiteSpace was used to analyze citations obtained by the Web of Science Core Collection (WoSCC) as a visualization tools (17). This software can provide great support for scientometrics research and fill the knowledge gap in bibliometric research of salvage surgery for HNC. The main purpose of this study is to: (1) provide a comprehensive and systematic review of research on salvage surgery for HNC in the past 30 years under the background of globalization at diverse structural levels of authors, institutions, countries, etc.; (2) study the hot research topics in this field and their characteristics; (3) summarize the overall development trend and characteristics based on trend analysis, and analyze the research direction with potential value.

## 2 Materials and methods

### 2.1 Research methods

Bibliometrics is a quantitative analysis method that uses various external characteristics of scientific documents as the research object, and uses mathematical and statistical methods to describe, evaluate, and predict the current status and development trends of science and technology. Bibliometric methodologies are good at exploring the potential knowledge structure contained in academic documents, such as keywords, references, etc., integrating and visualizing the results to further analyze the field (18). Large amounts of literature data can be analyzed using bibliometric software to generate visualized results (19).

CiteSpace, a software developed by Dr. Chen Chaomei of Drexel University in the United States and based on citation analysis theory, is an information visualization software that can present the structure, patterns, and distribution of scientific knowledge, with the resulting graphics called “scientific knowledge maps” (17). It is mainly used for sorting out theoretical perspectives, evolution paths, development trends, academic history, and hotspot scanning in scientific research fields (20). It is a practical literature analysis software for quantitative analysis. The analysis methods used in CiteSpace include co-citation analysis, co-occurrence analysis, burst detection, and clustering analysis (21). Among them, co-citation analysis is the analysis of the co-citation relationship between two studies appearing in a third study. The more times two studies are co-cited, the more similar they are and the more relevant they are to each other. Co-occurrence analysis counts the occurrences of a group of keywords in the literature of the research field and measures their affinity through their co-occurrence times. Burst detection can detect a decrease or increase in the used times of keywords. Clustering analysis groups objects based on their similarity and analyzes the multiple clusters formed (17, 22, 23). Centrality is a key indicator for analyzing the importance of objects. Nodes with intermediate centrality >0.1 are called central nodes, also known as key nodes, which are relatively important and have great influence in the research field and often play a role as bridges connecting different research objects such as articles, keywords, and countries (19). CiteSpace has unique advantages in identifying key points and future trends in research fields (17). Therefore, in this study, bibliometric analysis using CiteSpace was used. Another software, Vosviewer, is also used for periodical level analysis (24). In addition, critical reading was conducted to further analyze key research and provide major insights for this topic.

### 2.2 Data resource

**Figure 1:**
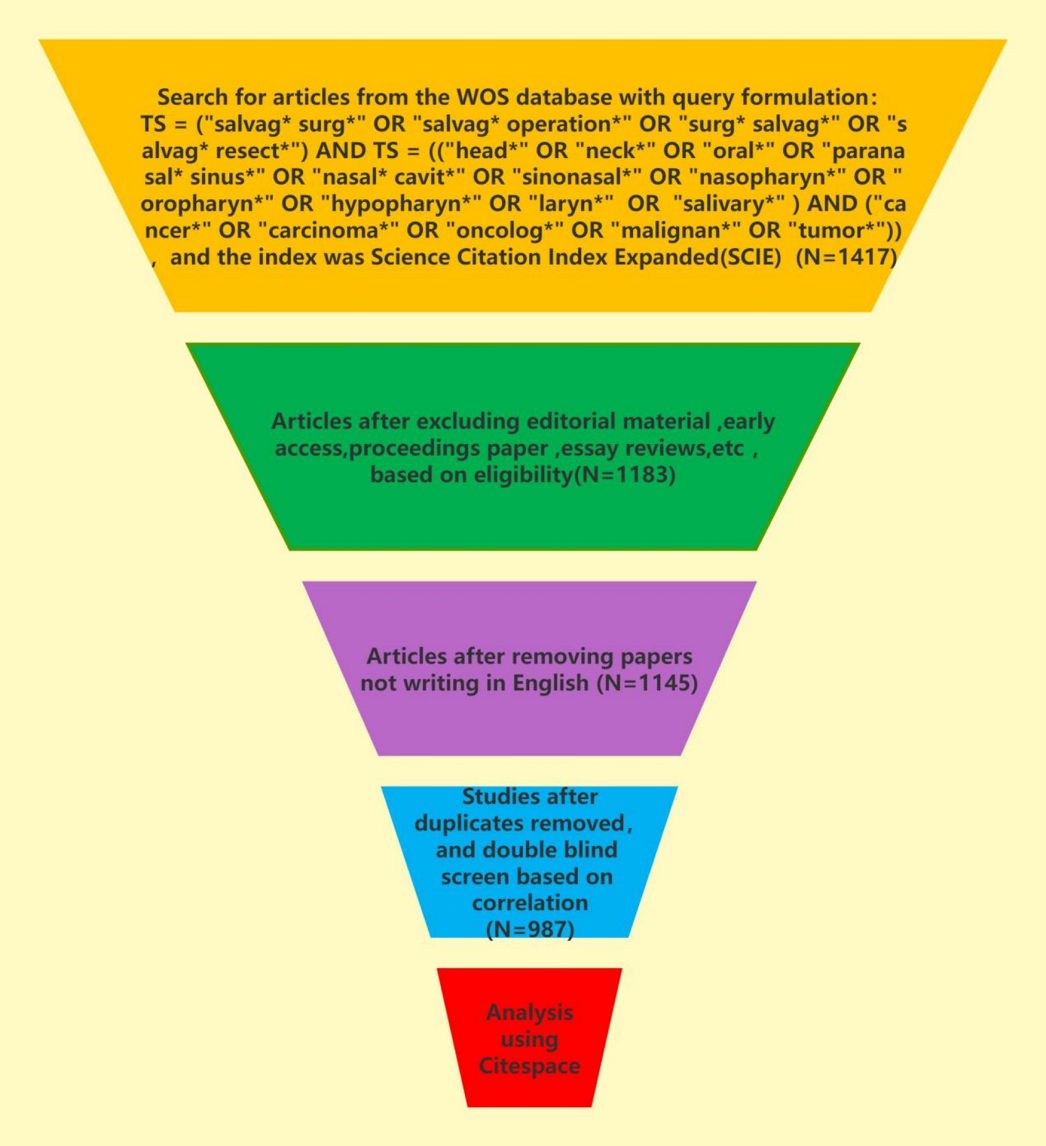
The literature retrieval and screening process of this study.

In this paper, Web of Science Core Collection (WoSCC) was selected and the index was Science Citation Index Expanded (SCIE). The retrieval formula in this paper was as follows: TS = (“salvag* surg*” OR “salvag* operation*” OR “surg* salvag*” OR “salvag* resect*”) AND TS = ((“head*” OR “neck*” OR “oral*” OR “paranasal* sinus*” OR “nasal* cavit*” OR “sinonasal*” OR “nasopharyn*” OR “oropharyn*” OR “hypopharyn*” OR “laryn*” OR “salivary*”) AND (“cancer*” OR “carcinoma*” OR “oncolog*” OR “malignan*” OR “tumor*”)). The time span was from January 1st, 2000 to December 31st, 2023. The time span was from January 1st, 2000 to December 31st, 2023. A total of 1,417 publications (1,183 articles, 164reviews, 44 conference abstracts and 26 others) were searched on January 1st 2024, and 1,145 articles were selected that are articles and writing in English after removing duplicates. We subsequently conducted a double-blind screening by correlation and identified the same subsets based on the following criteria: (1) the research subjects must be patients who have been diagnosed with HNC, but not other parts of the body; (2) the research subjects must be humans, not cats, dogs, pigs, or other animals; (3) the main focus of the research must be related to salvage surgery; (4) according to the WHO definition, the research site should exclude the eyes, thyroid, the brain and its surrounding tissues and so on. At last, this paper made a bibliometric analysis of the 987 articles. The literature retrieval and screening process was recorded in Figure 1 and Table 1.

**Table 1:** The search strategy used for the present bibliometric analysis.

Category	Specific standard requirements
Research database	Web of science core collection
Citation indexes	SCIE
Searching period	2000-01-01 to 2023-12-31
Language	“English”
Document types	“Articles”
Data extraction	Export with full records and cited references in plain text format
Query formulation	TS = (“salvag* surg*” OR “salvag* operation*” OR “surg* salvag*” OR “salvag* resect*”) AND TS = ((“head*” OR “neck*” OR “oral*” OR “paranasal* sinus*” OR “nasal* cavit*” OR “sinonasal*” OR “nasopharyn*” OR “oropharyn*” OR “hypopharyn*” OR “laryn*” OR “salivary*”) AND (“cancer*” OR “carcinoma*” OR “oncolog*” OR “malignan*” OR “tumor*”))
Sample size	987

## 3 Results

### 3.1 Analysis of publishing trend

**Figure 2:**
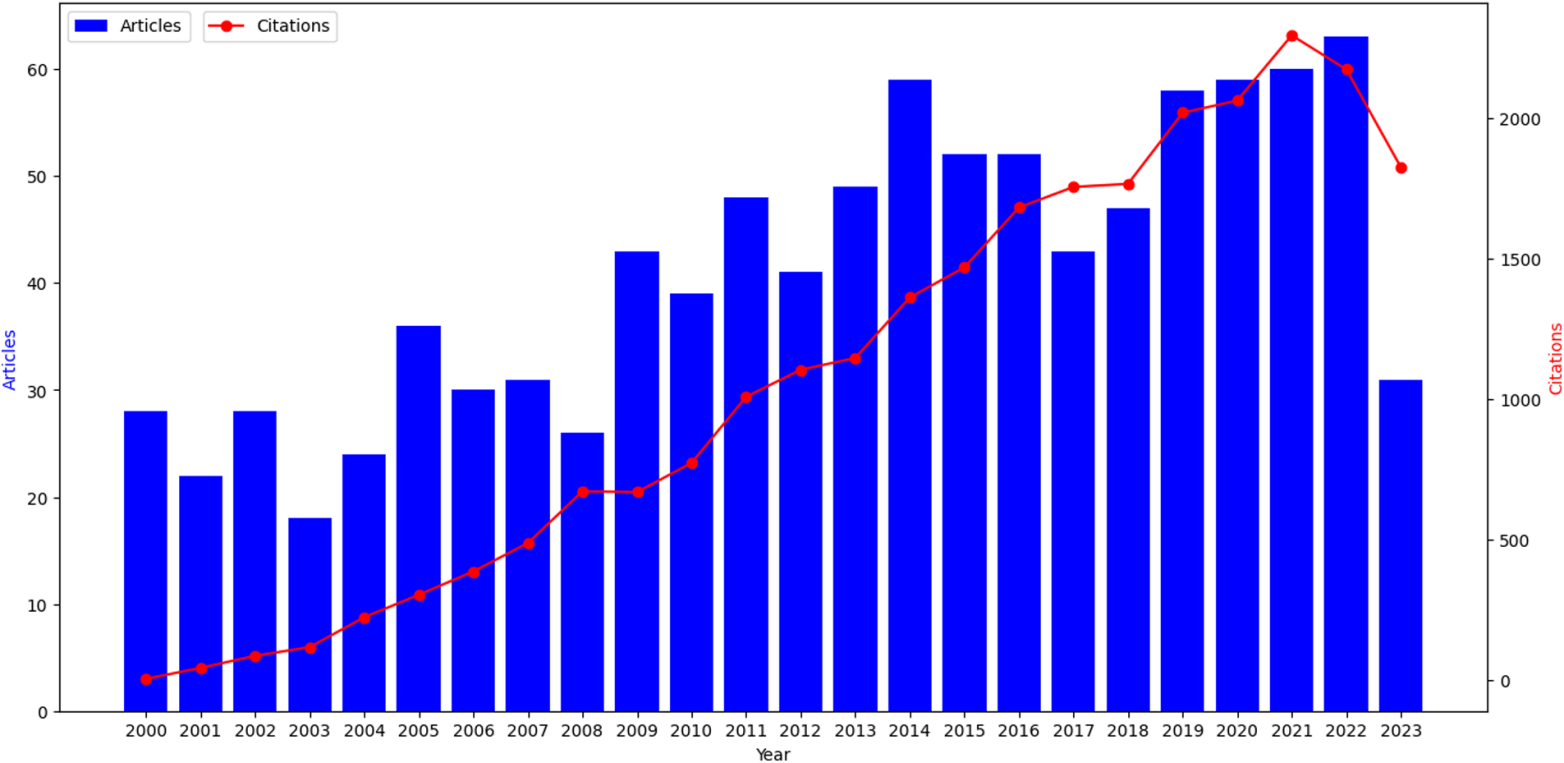
Time evolution of the total number of publications and citations in the WOS database. Time evolution of the total number of publications and citations in the WOS database is showed. The ordinate represents the number of published papers and citations, while the abscissa represents the years. The overall trend of the line chart shows an upward trend.

The trend of literature publication is an important index to measure the research development in a certain field. Therefore, by drawing the quantity-time curve of literature, we could effectively evaluate the research status in this field and further predict the development trend. Figure 2 showed the annual distribution of articles related to salvage surgery on Web of Science since the 21st century. It was calculated that the average number of articles was 41 per year since 21st century and the average citation count was 1,060.4. In summary, there has been significant growth and progress in the study of salvage surgery. The development of the whole research field could be divided into the first twelve years and the last twelve years according to the timeline. The annual number of articles published in the first twelve years was 31, with the annual number of 399 citations. In the last twelve years, the annual number of publication was 51, and the annual number of citations was 1,721. The total citation number has also increased from less than 6–1,825. Although the number of studies has not reached the highest level, the research on the salvage surgery has been developing rapidly. At the same time, according to the research trend, salvage surgery for HNC will still have a great developing in the future.

### 3.2 National, institutional, author and journal analysis

#### 3.2.1 National analysis

**Figure 3:**
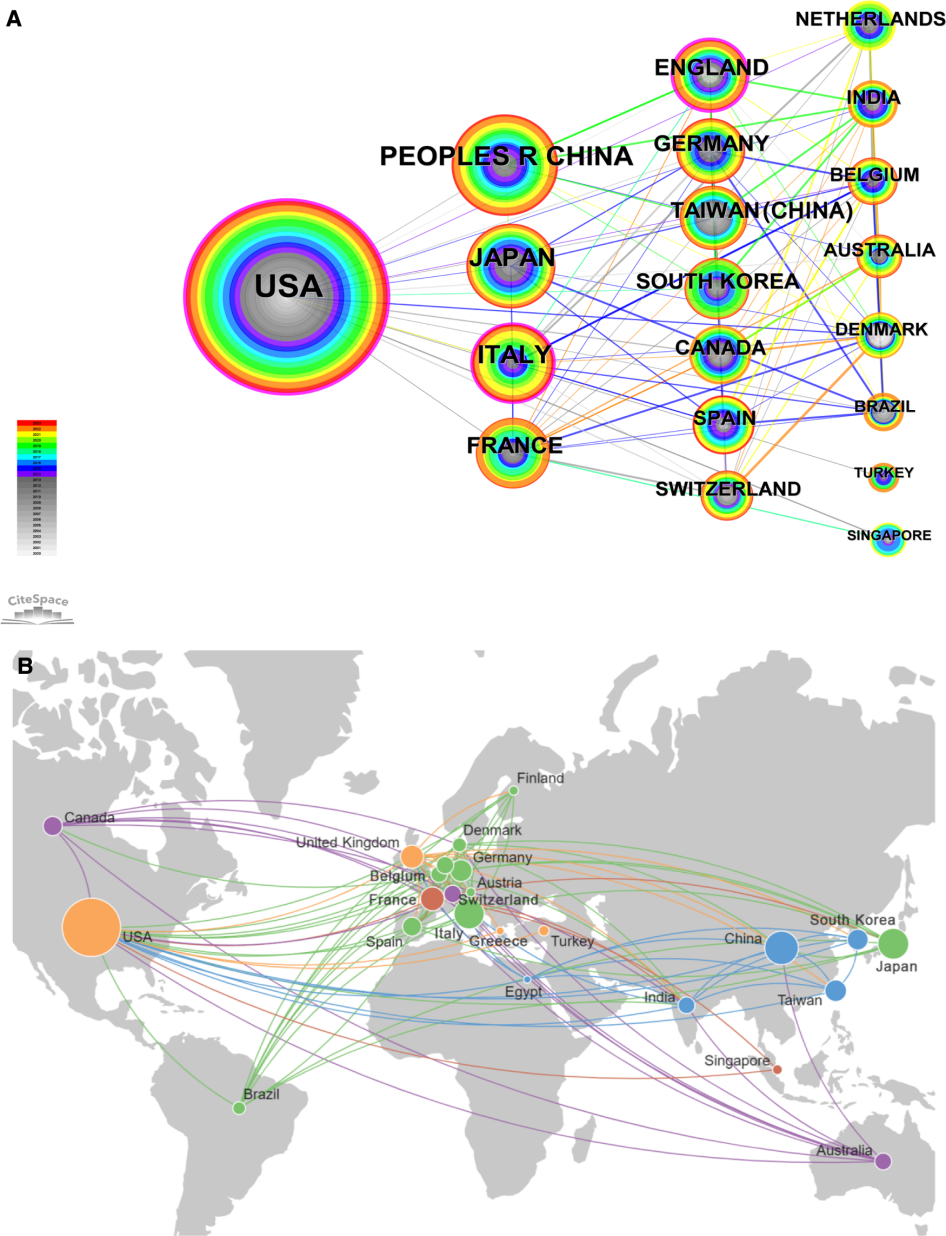
Analysis of publication among countries. (**A**) Cooperation network of countries. In the visualization, the purple outer ring represents countries with an intermediary centrality greater than 0.1. Intermediary centrality is a measure of the importance of the research object in the collaborative network, indicating the strength of its bridging role connecting multiple other countries. The size of the nodes corresponds to the volume of publications, with countries that have a higher number of publications being represented by larger nodes. (**B**) Analysis of geographical distribution of countries. This image in Citespace was created by connected to Google Maps. The size of the nodes in the visualization corresponds to the number of publications, with larger nodes indicating more publications from a country. To improve the visual appearance, connections between countries with fewer collaborations were left out.

Through the quantitative analysis at the aspect of countries, we could not only identify the core countries in the research field of salvage surgery for HNC, but also the academic communication and cooperation between countries in this field. In this study, the “Country” was selected as the analysis object in CiteSpace, the time “Time Slicing” was “2000–2023”, the “Years Per Slice” was “1”, and the threshold was top “20”. Finally, a national analysis map with 54 network nodes and 196 connections with a density of 0.137 was obtained, as shown in Figure 3. The thickness of the colored ring indicated the degree of its intermediate centrality, reflecting the importance of its position in the network. It could be seen in Figure 3 that more international connections suggested higher centrality and greater authority of a country.

The top 20 countries were listed in the Table 2. The frequency represented the count of publications in the country, and the centrality represented the importance of the country in this field. As can be seen from the frequency of Table 2, the USA had the most researches on salvage surgery for HNC, with 311 articles that were much more than other countries, indicating that the USA in the leading state has been very concerned about the research field of HNC-related salvage surgery. At the same time, the UK has been also the country with the highest intermediary centrality of ∼0.32, indicating that the transnational cooperation in this field was most common in UK. Although the number of publications was small, it has still been an important bridge connecting other countries in this field. The frequency of PEOPLES R CHINA ranked second, with 104 relevant studies published, and Japan ranked third, with 88 published. From the perspective of centrality, Chinese and Japanese scholars have always tended to study independently and lacked international exchanges. At the same time, Italy's intermediary centrality status also exceeded 0.1, indicating that Italy and Germany also had paid more attention to the research of HNC, and had good international cooperation. Almost all countries with an intermediary centrality greater than 0.1 were developed countries. Therefore, the developed countries have paid more attention to the research and international cooperation in this field. From the start time, we could know that the research in about half of the countries appeared at 2000, which meant that this field has received enough attention since the beginning of the 21st century.

**Table 2:** The centrality and count of literature in countries.

Country	Year	Frequency	Centrality
USA	2000	311	0.28
Peoples R China	2000	104	0.01
Japan	2000	88	0
Italy	2000	83	0.13
France	2001	52	0.03
United Kingdom	2000	49	0.32
Germany	2001	44	0.1
Taiwan	2000	43	0
South Korea	2001	40	0
Canada	2000	34	0.02
Spain	2000	33	0.07
Netherlands	2002	28	0.07
Switzerland	2000	28	0.06
India	2000	26	0.05
Belgium	2008	25	0.04
Australia	2002	25	0.02
Denmark	2000	18	0.01
Brazil	2001	15	0.04
Turkey	2005	10	0
Singapore	2005	9	0

“Year” denotes the initial publication year; “Frequency” indicates the number of publications; “Centrality” refers to intermediary centrality.

#### 3.2.2 Institutional analysis

**Figure 4:**
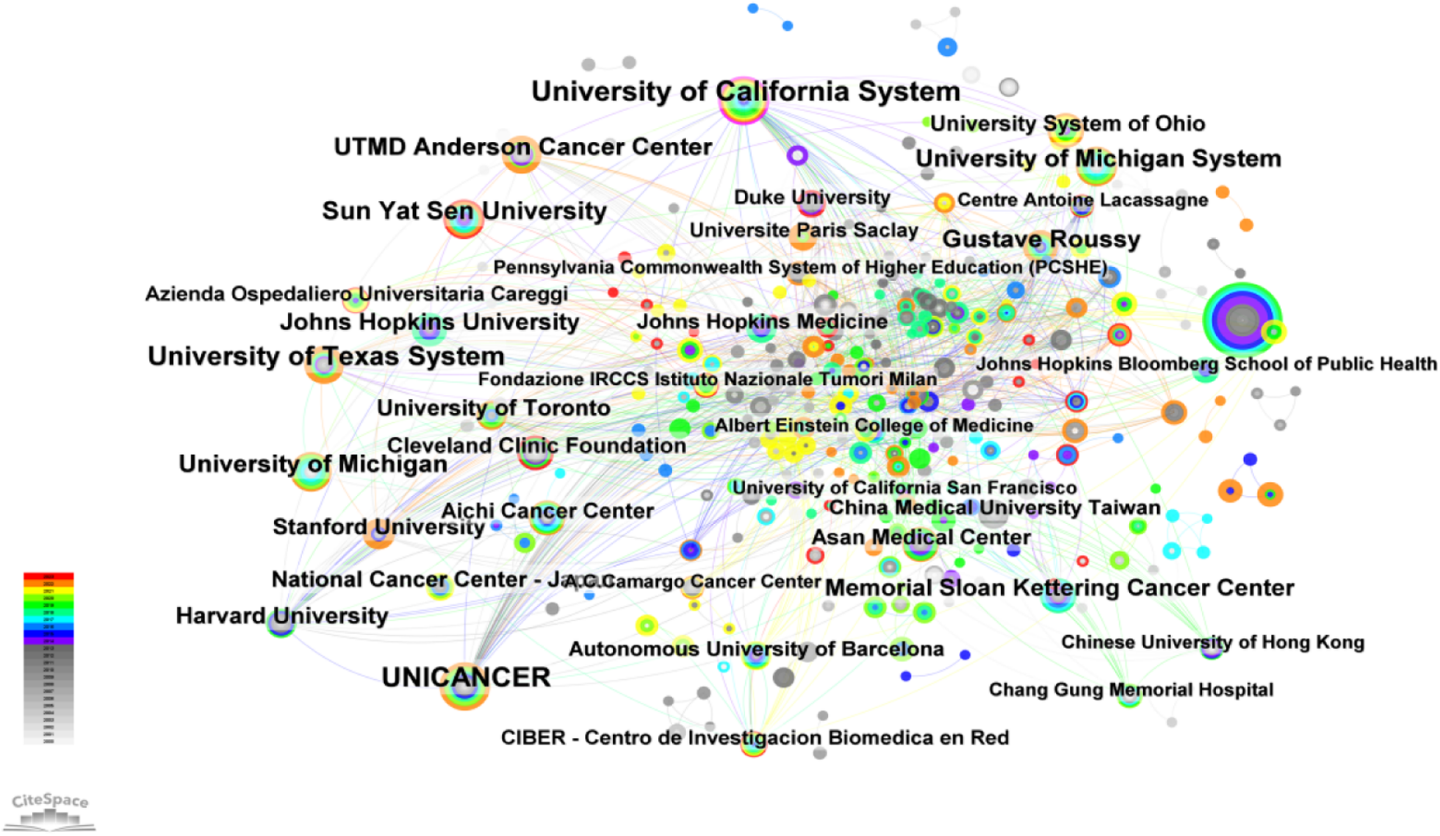
Inter-institutional cooperation network. Institutions with a purple outer ring indicate a centrality greater than 0.1. The size of the nodes corresponds to the frequency of article publications by each institution. To enhance the visual appeal of the image, the lines between institutions with relatively few collaborative instances have been omitted.

By using “Institution” in CiteSpace as the analysis object, an institutional analysis map with a density of 0.0129 with 425 network nodes and 1,162 connections was obtained. As shown in Figure 4, the nodes in the map were relatively dense and most of the data were connected with a total connection of 1,162, which indicated that institutional cooperation in the field of salvage surgery for HNC has been relatively frequent, and most research institutions had cooperation. Some of these institutional cooperation had obvious regional characteristics, which showed that inter-institution research needs to be maintained to promote academic exchanges in the field of salvage surgery for HNC.

This paper also collated the top 5 institutions in the field of salvage surgery for HNC. As can be seen from Table 3, University of California System, UNICANCER, University of Hong Kong were the top 3 institution, with a volume of 29, 27 and 26, leading other institutions. However, the centrality of University of Hong Kong was 0, which showed that University of Hong Kong had a relative higher frequency of publication, but less cooperation with other institutions. It can also be seen that the publication time of UNICANCER was earlier than others, indicating that UNICANCER has played a foundational role in the research of salvage surgery for HNC.

**Table 3:** The top 5 institutions of publication.

Institution	Year	Frequency	Centrality
University of California System	2007	29	0.17
UNICANCER	2001	27	0.09
University of Hong Kong	2003	26	0
University of Texas System	2004	23	0.07
Sun Yat Sen University	2009	19	0.01

“Year” denotes the initial publication year; “Frequency” indicates the number of publications; “Centrality” refers to intermediary centrality.

Although the Sun Yat Sen University paid attention to the research field of salvage surgery for HNC relatively later, its number of articles published has also been relatively higher. The institution with the highest intermediary centrality has been University of California System, which was also the institution with the highest number of publications. It could be speculated that stronger institutional cooperation was conducive to the generation of scientific results.

#### 3.2.3 Author cooperation analysis

**Figure 5:**
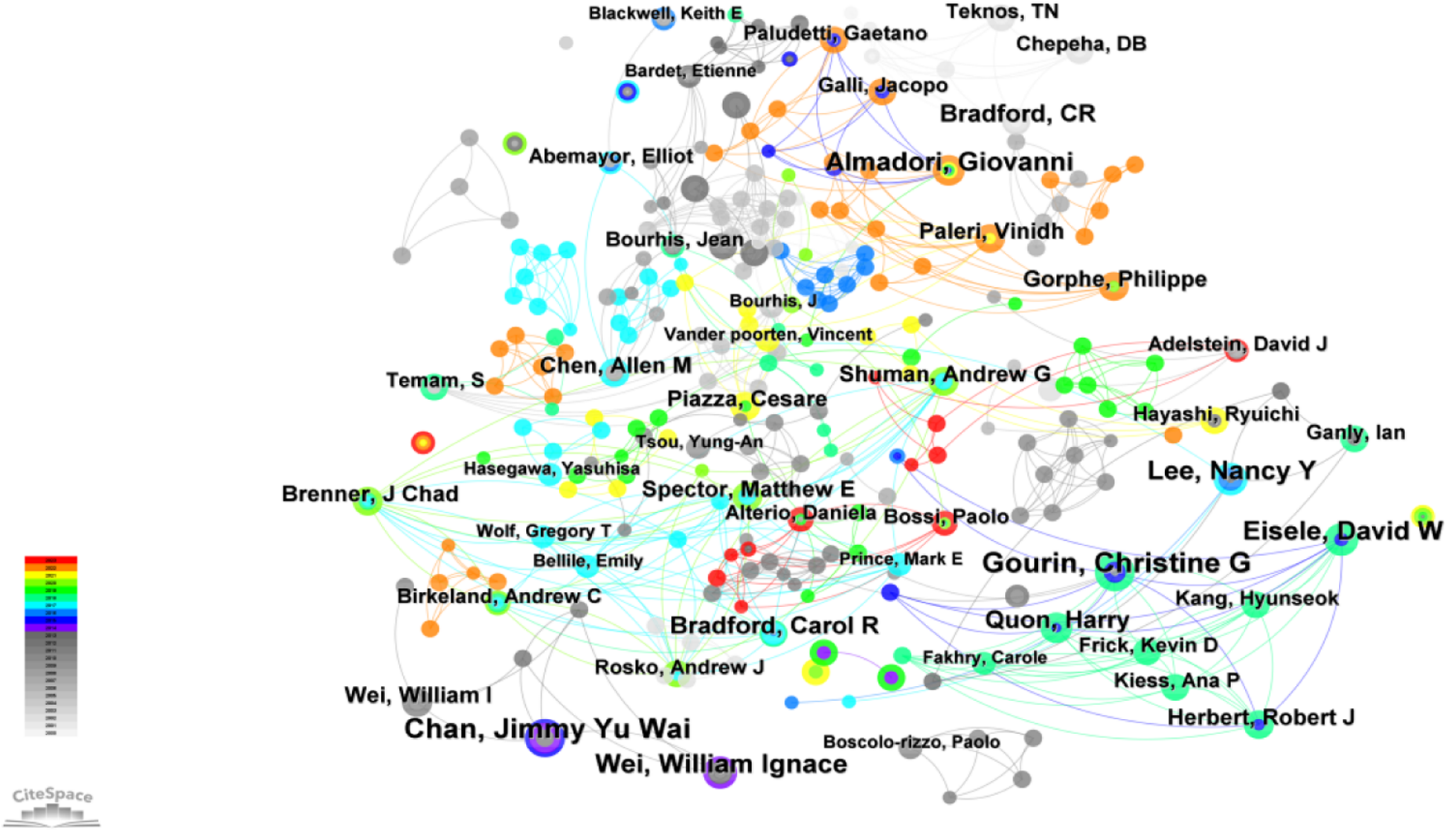
Analysis diagram of author collaboration network. The size of the nodes corresponds to the frequency of article publications by each author. The lines between institutions with relatively few collaborative instances have been omitted.

Based on the analysis of “authors” in CiteSpace, an author analysis map with 705 network nodes, 886 connections, and a density of 0.0036 was obtained (Figure 5). In this map, the three largest nodes are Gourin, Christine G; Chan, Jimmy Yu Wai and Eisele, David W, with publication counts of 9, 9, and 7, respectively. This indicated that they have been the most important authors in this field. We could see from the Figure 5 and Table 4 that none of the authors had produced more than 10 papers in this research field. There have been multiple researchers, but the average output has been relatively less, indicating that the directions of the field have been very dispersed.

**Table 4:** Top 10 authors in publication count.

Frequency	Burst	Begin	End	Authors
9	0	None	None	Gourin, Christine G
9	4.89	2012	2015	Chan, Jimmy Yu Wai
7	3.34	2015	2018	Eisele, David W
7	0	None	None	Almadori, Giovanni
7	3.25	2013	2017	Lee, Nancy Y
7	4.11	2012	2014	Wei, William Ignace
6	0	None	None	Bradford, Carol R
6	0	None	None	Quon, Harry
6	3.44	2000	2003	Bradford, CR

“Year” denotes the initial publication year; “Frequency” indicates the number of publications.

#### 3.2.4 Journal co-citation analysis

**Figure 6:**
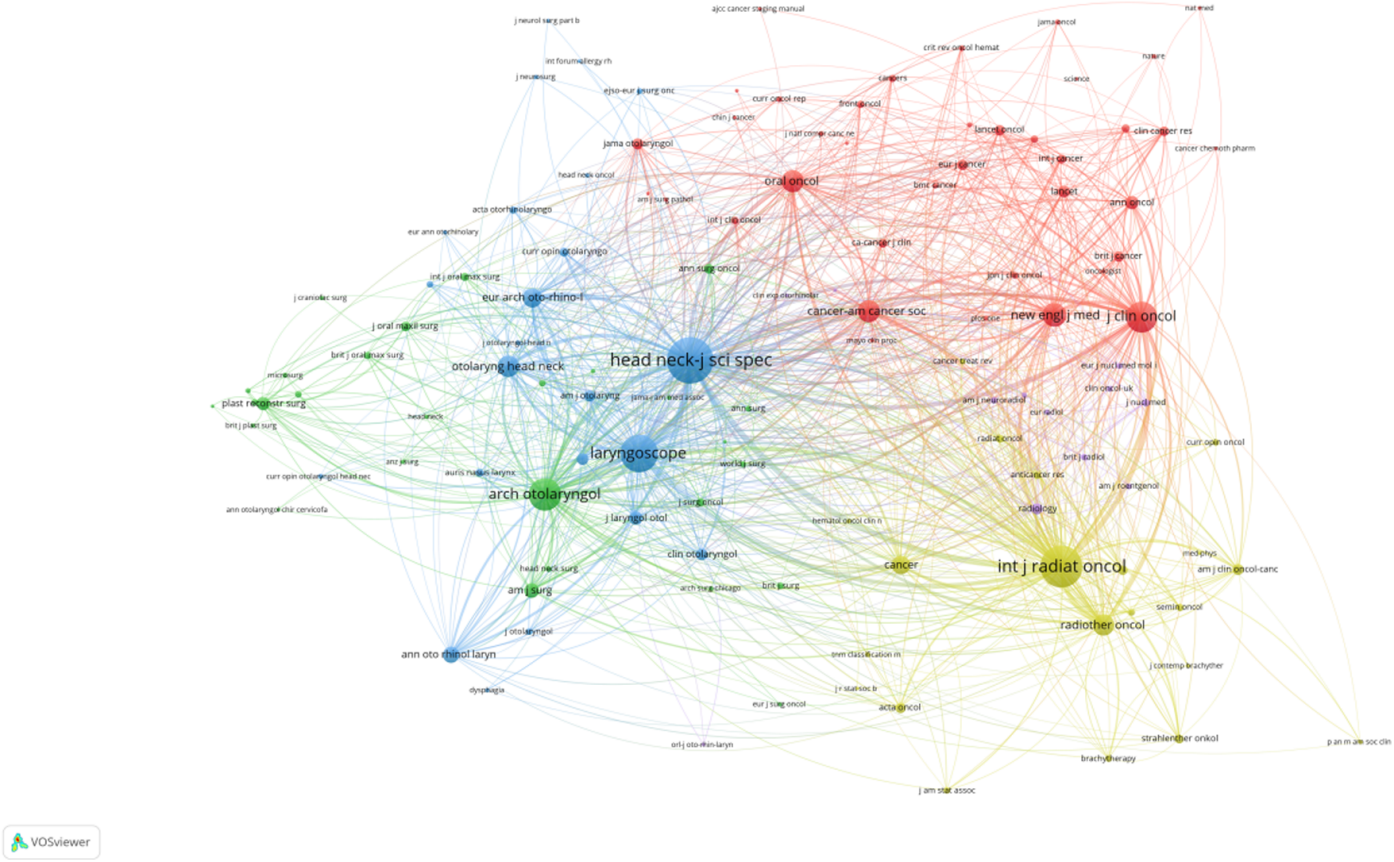
Analysis diagram of journal co-citation network. Each node represents a journal, with larger nodes indicating a greater number of published articles. Journals of the same color are indicative of closely related topics in their published articles, and the lines connecting the nodes represent their co-appearance in citations within the same articles.

After statistical analysis of publications and journal citations using VOSvier (Figure 6), we identified the three most authoritative core journals in this field. By observing the node size in Figure 6, we could intuitively see the journals that published the most articles in this field. Table 5 listed the top 10 journals in the field with citation counts. From the comparison of journal publications, citations and impact factors, the core journals of salvage surgery for HNC were identified as *Head And Neck-Journal For The Sciences And Specialties Of The Head And Neck*, *International Journal of Radiation Biology*, and *Laryngoscope*. *Head And Neck-Journal For The Sciences And Specialties Of The Head And Neck* has published the most articles of salvage surgery for HNC and achieved the highest citations, making it an undeniable core journal. It was an international multidisciplinary publication of original contributions concerning the diagnosis and management of diseases of the head and neck, involving the overlapping interests and expertise of several surgical and medical specialties. *International Journal of Radiation Biology* covered topics that range from radiation chemistry to the spectrum of responses of living organisms and underlying mechanisms, addressing research on the application of basic studies for the medical uses of radiation. The *Laryngoscope* was also one of the leading sources about information on advances in the diagnosis and treatment of head and neck disorders. *Journal of Clinical Oncology*, *Cancer*, and *International Journal of Radiation Biology* had the highest numbers of citations per article, which were 166, 71, and 63 respectively, indicating that their published articles had a greater influence in the field.

**Table 5:** Journals with the Top10 citation numbers.

Rank	Journals	Publications	Number of citations
1	Head And Neck-Journal For The Sciences And Specialties Of The Head And Neck	116	3,274
2	International Journal of Radiation Biology	44	2,800
3	Laryngoscope	76	2,549
4	Archives of Otolaryngology—Head & Neck Surgery	38	1,779
5	Cancer	22	1,579
6	Journal of Clinical Oncology	9	1,496
7	Oral Oncology	40	898
8	European Archives Of Oto-rhino-laryngology	50	829
9	Otolaryngology–Head & Neck Surgery	39	790
10	Radiotherapy and Oncology	15	714

“Documents” represents the number of articles published in the journal; “Citations” refers to the total number of citations received by the relevant articles published in the journal; “Average Citations” indicates the average number of citations received by articles in the journal.

### 3.3 Keywords cluster, frontiers, trend, and literature co-citation analysis

#### 3.3.1 Keywords cluster and co-citation analysis

**Figure 7:**
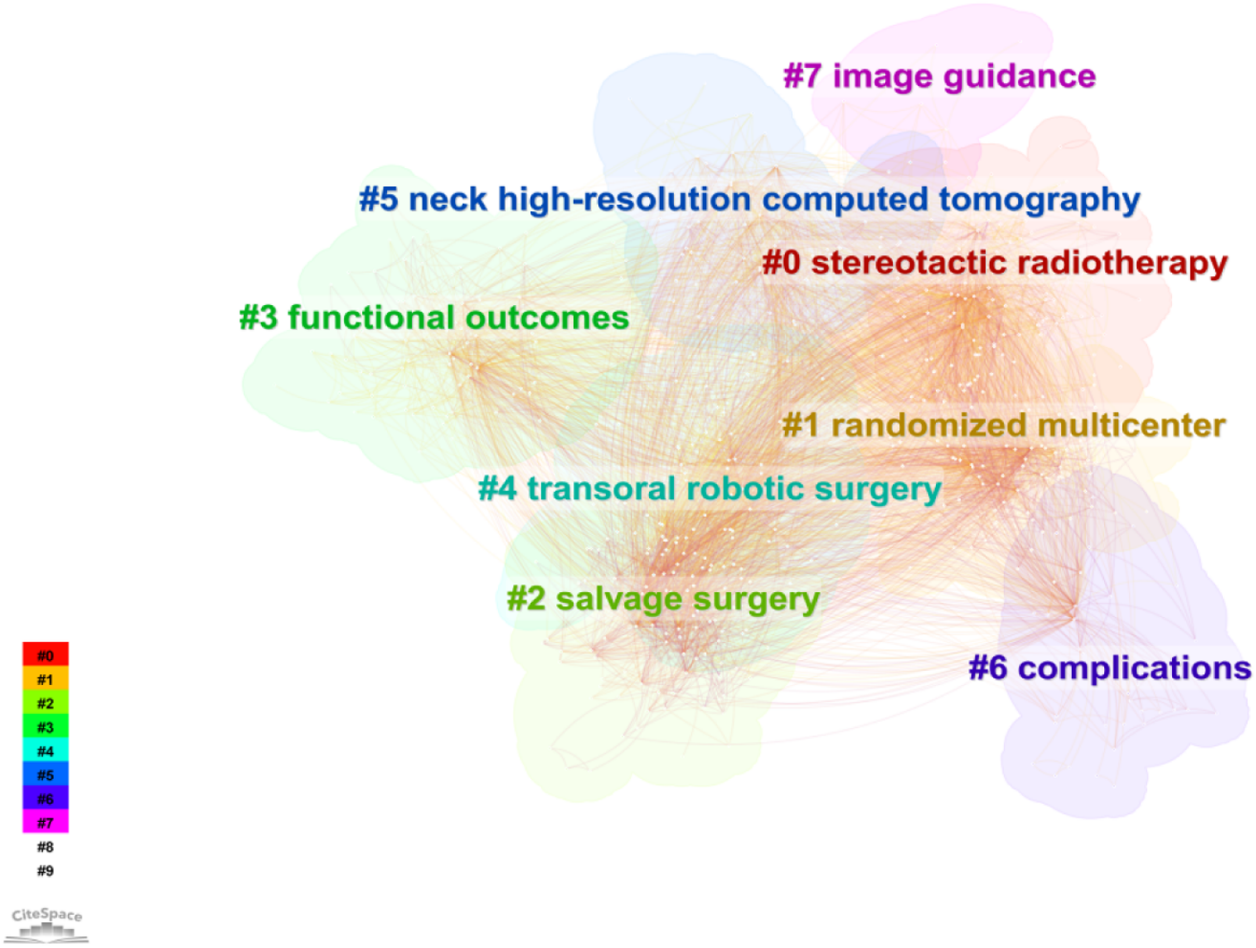
Analysis of keywords clustering. This figure was made by selected the “cluster” option, and used the panthfinder algorithm to cut the connection lines to ensure the classification rationality of clusters. A total of 8 keyword clusters have been identified, with each cluster assigned a different color based on the time in the bottom left corner. The cluster names were derived from a set of representative keywords.

We used CiteSpace to cluster the keywords, selected the “cluster” option, and used the panthfinder algorithm to cut the connection lines to ensure the classification rationality of clustering. The results were shown in Figure 7, which reflected the research topics in the field of salvage surgery of HNC since 2000. A total of 8 clusters were obtained. As can be seen from Figure 7, this study generated a total of 8 clusters, respectively: #0 stereotactic radiotherapy (2012); #1 randomized multicenter (2007); #2 salvage surgery (2004); #3 functional outcomes (2014); #4 transoral robotic surgery (2013); #5 neck high-resolution computed tomography (2010); #6 complications (2008); #7 image guidance (2019).

There was an obvious developing trail in the field of salvage surgery for HNC. The initial cluster was the preliminary development of salvage surgery, such as the “salvage surgery” clustering in 2004, and then the study began to focus on the complications of salvage surgery (2008). Since the salvage surgery of HNC has involved a variety of parameters for preoperative evaluation, prognosis, and many other issues that need to be studied and determined, “random multi-center” has also become the focus of research during this period. With the advancement of high-resolution computed tomography technology, CT has become an important auxiliary examination tool for preoperative evaluation and perioperative period of salvage surgery for HNC (2010). In the field of salvage therapy for HNC, more and more studies have begun to compare the advantages and disadvantages of stereoscopic quantitative radiotherapy and surgery for patients and their applicable occasion (2012). The application of robotic surgery in the field of HNC has received more and more attention (2013), which have greatly improved the accuracy of surgery. Studies in later period began to pay more attention to patients' functional outcomes and quality of life (2014). Salvage surgery of HNC guided by image technology has been a hot spot in recent years, which was helpful to reduce complications, improve accuracy and expand the scope of application.

#### 3.3.2 Analysis of research frontiers

**Figure 8:**
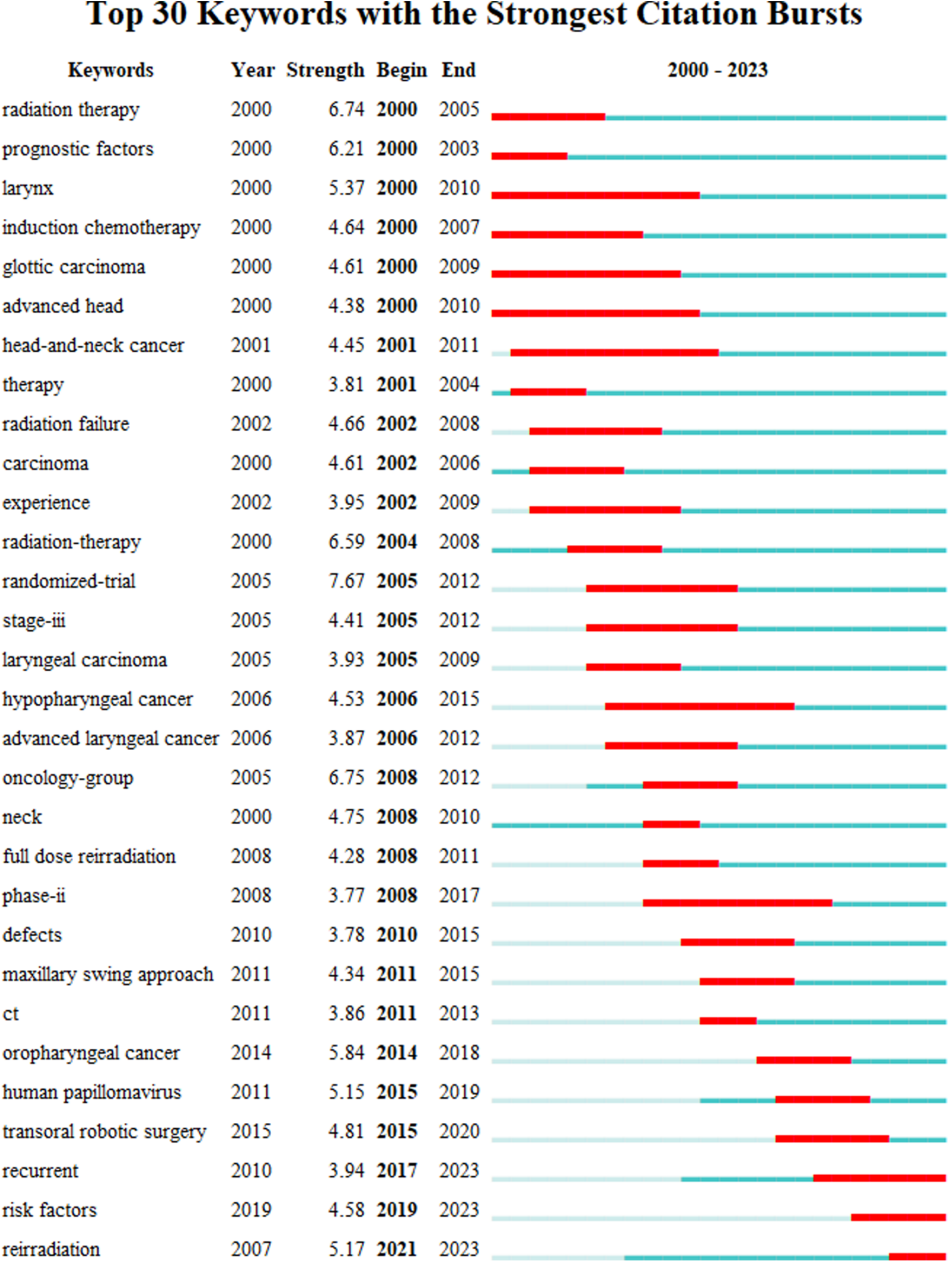
Top 30 keywords with the strongest citation bursts. The Bursts detection algorithm of Citespace software was used to obtain the hot spot evolution map in the field on Web of Science. This study generated the top 30 burst keywords with their burst intensity and duration in the field. The timespan is indicated by a blue line. The time period during when an outbreak appeared is displayed as a red segment, indicating the start and end years of the outbreak duration. “Year” in the figure represents the year when the keyword first appeared. “Strength” indicates the intensity of the keyword outbreak.

The bursts detection algorithm of CiteSpace was used to obtain the hot spot evolution map in the field of salvage surgery for HNC research on Web of Science, that is, burst keywords. As shown in Figure 8, this study generated the top 30 burst keywords with their burst intensity and duration in the field. The timespan was indicated by a blue line. The time period when an outbreak appeared was displayed as a red segment, indicating the start and end year of the outbreak duration.

According to the burst beginning time, the study could be divided into two parts: before 2011 and after 2011. The former had 24 burst keywords, which were very much more than that of the later period and lasted for a long time about 6.8 years. The number of burst keywords after 2011 was fewer, and the duration of the outbreak was also shorter, which was 5.2 years, suggesting that the speed of hot spot change was faster in the later period, and there have been many mature researches in lots of directions in the field. In addition, at present, the hottest keywords were “recurrent”, “risk factors”, “reirradiation”.

We found that in the initial stage, the study focused on the relationship between salvage surgery and chemoradiotherapy for HNC, and emphasized the importance of doctor experience in the salvage treatment of HNC in 2002. Then in 2005, “clinical trial” and “trial” became the focus of research, and scholars paid more and more attention to the level of clinical evidence. The occasion of surgery and preoperative comprehensive evaluation has been paid a lot of attention, such as keywords “stage-3”, “oncology group”, “risk factor”, etc. Afterwards, the impact of HPV on HNC and salvage surgery has begun to receive attention. In addition, the application of robotic surgery in salvage surgery has significantly improved the accuracy of surgery.

#### 3.3.3 Trends analysis

**Figure 9:**
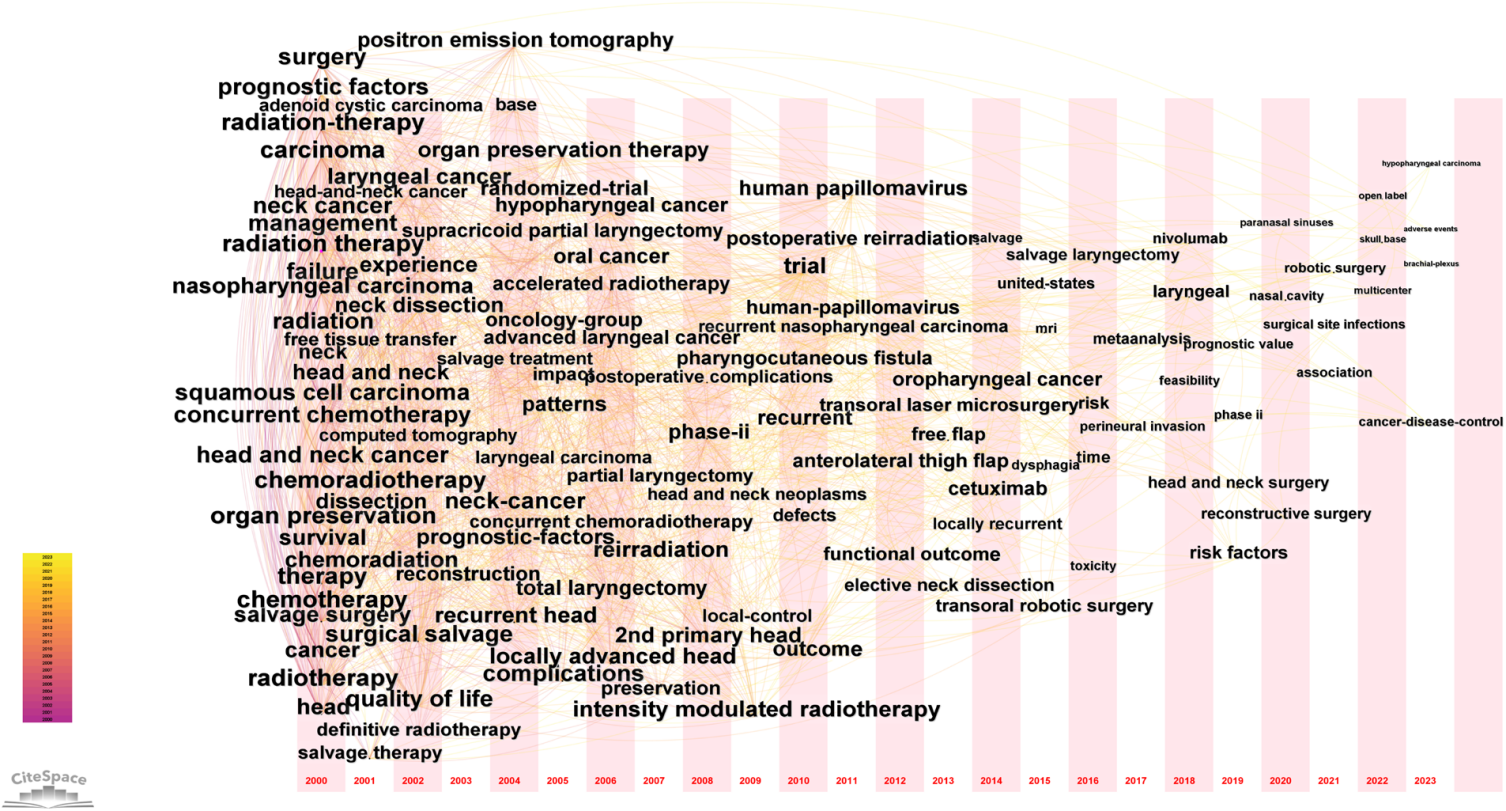
The timezone chart of keywords in region of salvage surgery for HNC. The keywords timezone map of the domain was generated by selecting “Timezone” as the analysis node through Citespace. The specific indicators and thresholds were set as follows: the time slice was “1”, the keywords with smaller nodes were hidden, and the current graph was generated, as shown in the figure.

The keywords timezone map of the domain was generated by selecting “Timezone” as the analysis node through CiteSpace, that is, the changes of keywords with time. The specific indicators and thresholds were set as follows: the time slice was “1”, and the current graph was generated, as shown in the Figure 9. The time zone diagram described the change of keywords over time. The time slice corresponding to the keyword was the year in which the keyword first appeared.

It could be seen from most of the keywords that in the first 5 years (2000–2004), the debate on salvage surgery, radiotherapy, chemotherapy, and organ preservation have begun to be presented by many scholars in the field. Moreover, more emphasis was placed on the role of doctors' personal experience in salvage surgery for HNC, such as the keyword “experience”. In the next ten years (2005–2014), scholars began to pay attention to the collection and exploration of clinical evidence for salvage surgery for HNC, such as keywords “trial”, “randomized-trial”, etc. Besides, many aspects of salvage surgery have begun to be fiercely explored, such as the occasion of treatment, as shown by the keywords “phase-2” and “local-contral”. A variety of surgical methods of salvage surgery have also be clarified, such as “transoral laser microsurgery”, “partial nephrectomy”, “supracricoid partial nephrectomy”, etc.

The emergence of cetuximab in 2014 indicated that salvage surgery after immunotherapy has also been explored. In addition, the relationship between HPV and HNC and its impact on salvage surgery have also been extensively explored. The postoperative complications and functional results of salvage surgery have also been concerned, such as “functional outcome”, “complications”, “prognostic_factors”, “defects”, etc.

In the last decade (2015–2023), robots began to appear in the field of salvage surgery and were continuously studied, such as the emergence of the keywords “robtic surgery” and “transoral robotic surgery”. In addition, the level of clinical evidence has been getting higher and higher, and keywords such as “mataanalysis” and “multicenter” have emerged. The emergence of “nivolumab” indicated that immunotherapy combined with salvage surgery has been further developed.

#### 3.3.4 Literature co-citation analysis

**Figure 10:**
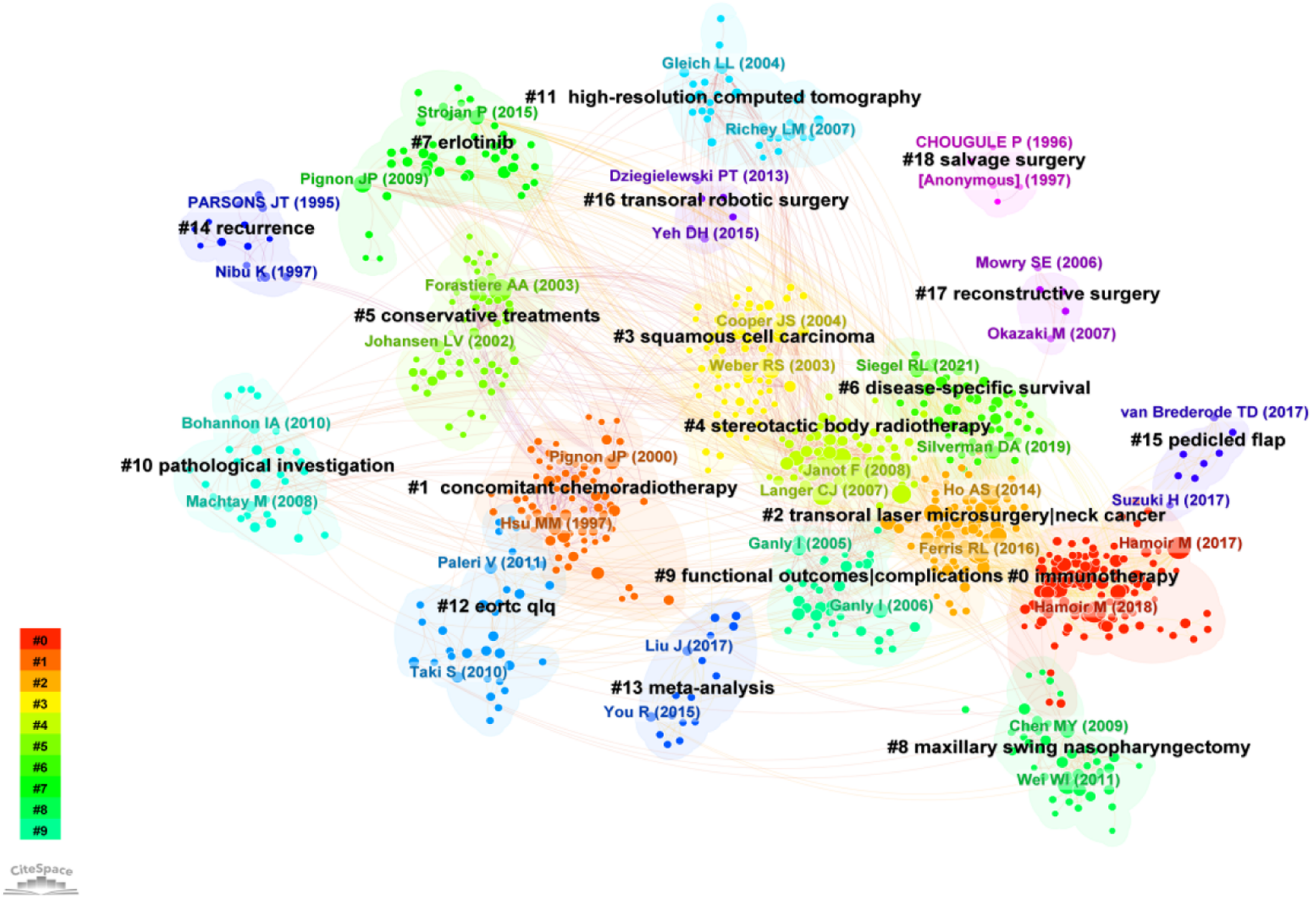
Clustering by the co-citation analysis. The co-citation analysis divided the literature in the field of HNC salvage surgery into 19 clusters.

The co-citation analysis divided the literature in the field of HNC salvage surgery into 19 clusters (Figure 10): #0 immunotherapy (2018), #1 concomitant chemoradiotherapy (1998), #2 transoral laser microsurgery|neck cancer (2014), #3 squamous cell carcinoma (2002), #4 stereotactic body radiotherapy (2007), #5 conservative treatment (2001), #6 disease-specific survival (2018), #7 erlotinib (2012), #8 maxillary swing nasopharyngectomy (2010), #9 functional outcomes|complications (2005), #10 pathological investigation (2009), #11 high-resolution computed tomography (2007), #12 eortc qlq (2012), #13 meta-analysis (2016), #14 recurrence (1996), #15 flap (2016), #16 transoral robotic surgery (2014), #17 reconstructive surgery (2006), #18 salvage surgery (1996). Among them, clustering 0, 1, 7, and 14 focused on the treatment for patients or state of disease before the implementation of salvage surgery. It was noteworthy that salvage surgery following immunotherapy has been a hot research point in recent years. Clustering 2, 8, 16, and 18 focused on the surgical methods of salvage surgery. In the past decade, robotic surgery and microsurgery have been widely used, greatly improving the accuracy. Cluster 3, 10, and 11 have been important indicators for preoperative comprehensive evaluation of salvage surgery. Cluster 6, 9, 15, and 17 focused on the prognosis, complications and their related solutions of salvage surgery. Due to the large number of clinical situations and directions in this field, meta-analysis has been conducive to obtaining the most favorable evidence and improving the level of evidence-based.

The greater the centrality of the intermediary, the more times the article appears with other articles. The diverse connections of the article with other themes and directions suggested the important position and significance of the article. In this topic, there were 20 articles with intermediary centrality greater than 0.1. The main contents were the clinical trials and reviews that clarified the selection of HNC patients to receive salvage surgery, and the prognosis of the patients that underwent salvage surgery. The efficacy of organ-sparing chemoradiotherapy was compared and other treatment methods combined with salvage surgery were also explored. The article with the highest centrality was Zafereo ME's “the role of salvage surgery in patients with recurrent squamous cell carcinoma of the oropharynx”, reaching 0.29. The objective of this study was to comprehensively review overall survival, functional outcomes, and prognostic factors of patients with salvage surgery as treatment for locally recurrent oropharynx squamous cell carcinoma after initial radiotherapy.

## 4 Discussion

This study conducted a comprehensive bibliometric research in the field of salvage surgery for HNC. We also studied the characteristics of HNC-related salvage surgery research from multiple perspectives, including publication characteristics, countries, institutions, author collaboration, core journals, field development trends, and research hotspots, etc. Since the 21st century, there have been 987 journal articles related to this field, and the number of articles has steadily increased, indicating that researchers have paid more attention to this field. In addition to analyzing and discussing the results of this study, we also conducted a critical review to better illustrate the topic of this research.

Due to many uncertain issues involved in salvage surgery for head and neck tumors, such as preoperative evaluation parameters and prognosis, people have shifted from trusting doctors' experience to pursuing the level of clinical evidence. With the development of high-resolution computerized tomography technology, imaging tools such as CT have become important auxiliary examination tools for preoperative evaluation and perioperative management of salvage surgery for head and neck tumors. More and more studies are comparing the advantages and disadvantages of three-dimensional quantitative radiation therapy and surgery, as well as their suitable occasion. Robotics and image-guided technology have been hot topics of research in the field of HNC, helping to reduce complications, improve accuracy, and expand application scope. With the emergence of cetuximab, rescue surgery after immunotherapy has also been explored, and the relationship between HPV and rescue surgery for HNC has also been of concern.

Salvage surgery for HNC has been still flourishing. Therefore, we need more research and continuous observation on this topic to see whether research institutions and researchers in different countries would have more in-depth and cutting-edge views on such topics. This means that despite these encouraging results, there are still problems in this study. It is important to acknowledge 5 primary limitations of this study:

1. The relatively limited volume of available literature in this specialized field, as we retrieved and screened literature using a double-blind method. This may lead to certain years lacking sufficient articles with threshold keywords, authors, institutions, or countries.
2. The constraints of bibliometric analysis may hinder a comprehensive description of all facets within this research domain. Due to software limitations, we only analyzed articles and reviews as they have complete citation records. CiteSpace's co-citation and co-occurrence functions can only analyze documents with complete records (17), and the above-mentioned studies are more detailed and reliable compared to other types.
3. Due to software design considerations, the visual analysis results may not effectively capture newly published high-quality literature when compared to older studies (17).
4. Given that we used TS retrieval, rather than full-text retrieval, there is a possibility of missing some relevant literature. However, full-text retrieval results in a very high number of articles, leading to a significant increase in unnecessary manual screening efforts.

Efforts will be made in the future to address these limitations and enhance the accuracy of trend forecasting. In future articles, we can utilize a variety of bibliometric analysis tools, such as R language, Citexs, and other similar tools. We can also combine full-text retrieval with AI screening technology to save manpower and increase the number of relevant articles (25). Regarding the time constraints on literature, we can limit it to the past decade or 20 years. For newly published, less-cited, high-quality literature, we can employ manual reading or write a review to highlight their importance.

## 5 Conclusion

The number of literature on salvage surgery for HNC has been increasing year by year, with a rapid increase in the average annual citation count. Most important documents have been published in high-impact journals. The United States published 311 articles with the highest publication volume, while the UK had the highest intermediary centrality. The most central institution is the University of California system, which has also consistently been the top institution in terms of publication volume. The average author's publication volume has not exceed ten articles. *Head And Neck-Journal For The Sciences And Specialties Of The Head And Neck* has published the highest number of articles and achieved the highest citations, acting as the undeniable core journal. The most advanced hotspots identified from comprehensive analysis comprises image-guided technology, CT, robotic surgery, meta-analysis, HPV, immune modulation, etc.

## Data Availability

The original contributions presented in the study are included in the article/Supplementary Material, further inquiries can be directed to the corresponding author.
